# Methyl 6-de­oxy-6-iodo-2,3-*O*-iso­propyl­idene-α-d-manno­pyran­oside

**DOI:** 10.1107/S1600536813022629

**Published:** 2013-08-17

**Authors:** Zeynep Gültekin, Wolfgang Frey, Nagihan Çaylak Delibaş, Tuncer Hökelek

**Affiliations:** a Department of Chemistry, Çankırı Karatekin University, TR-18100, Çankırı, Turkey; b Universität Stuttgart, Pfaffenwaldring 55, D-70569 Stuttgart, Germany; c Department of Physics, Sakarya University, 54187 Esentepe, Sakarya, Turkey; d Department of Physics, Hacettepe University, 06800 Beytepe, Ankara, Turkey

## Abstract

In the title compound, C_10_H_17_IO_5_, the six-membered tetra­hydro­pyran ring and the five-membered 1,3-dioxolane ring adopt sofa and envelope conformations, respectively. In the crystal, O—H⋯O and C—H⋯O hydrogen bonds link the mol­ecules into layers nearly parallel to the *bc* plane.

## Related literature

For carbohydrates which are important for the preparation of unsaturated aldehydes, see: Kleban *et al.* (2000 ▶); Dransfield *et al.* (1999 ▶); Greul *et al.* (2001 ▶). For conversions of unsaturated aldehydes to oximes, nitro­nes and nitrile oxides, see: Dransfield *et al.* (1999 ▶); Bernet & Vasella (1979 ▶); Greul *et al.* (2001 ▶); Gallos *et al.* (1999 ▶); Kleban *et al.* (2001 ▶). For the methods reported in the literature for the preparation of the title compound, see: Garegg & Samuelsson (1980 ▶); Bundle *et al.* (1988 ▶); Ichikawa *et al.* (2004 ▶). For the synthesis of methyl 2,3-*O*-iso­propyl­idene-α-d-manno­pyran­oside, see: Evans & Parrish (1977 ▶); Isobe *et al.* (1981 ▶). For ring-puckering parameters, see: Cremer & Pople (1975 ▶).
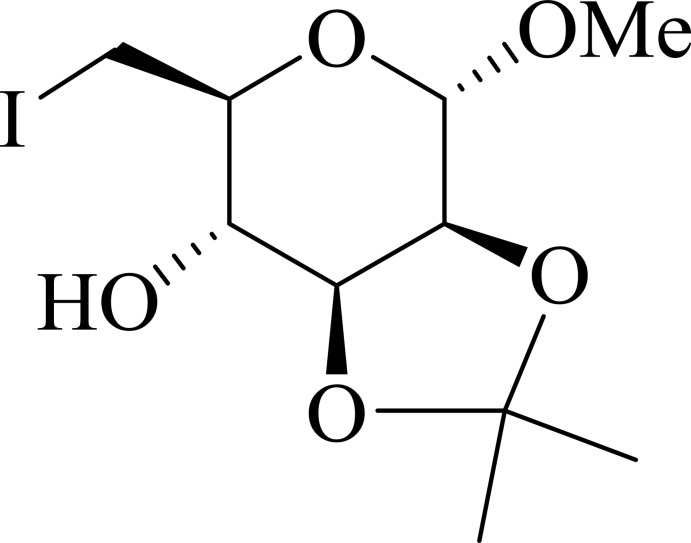


## Experimental

### 

#### Crystal data

C_10_H_17_IO_5_
*M* *_r_* = 344.14
Monoclinic, 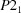

*a* = 8.3121 (8) Å
*b* = 10.3911 (10) Å
*c* = 8.3128 (8) Å
β = 118.639 (3)°
*V* = 630.15 (11) Å^3^
*Z* = 2
Mo *K*α radiation
μ = 2.55 mm^−1^
*T* = 100 K
0.99 × 0.58 × 0.44 mm

#### Data collection

Bruker Kappa APEXII DUO diffractometer
Absorption correction: numerical (Blessing, 1995 ▶) *T*_min_ = 0.187, *T*_max_ = 0.401
13656 measured reflections
3827 independent reflections
3803 reflections with *I* > 2σ(*I*)
*R*_int_ = 0.027
Standard reflections: 0

#### Refinement

*R*[*F* ^2^ > 2σ(*F* ^2^)] = 0.016
*wR*(*F* ^2^) = 0.043
*S* = 1.24
3827 reflections
153 parameters
3 restraints
H atoms treated by a mixture of independent and constrained refinement
Δρ_max_ = 0.86 e Å^−3^
Δρ_min_ = −0.82 e Å^−3^
Absolute structure: Flack (1983 ▶), 1811 Friedel pairs
Absolute structure parameter: 0.003 (12)

### 

Data collection: *APEX2* (Bruker, 2008 ▶); cell refinement: *SAINT* (Bruker, 2008 ▶); data reduction: *SAINT*; program(s) used to solve structure: *SHELXS97* (Sheldrick, 2008 ▶); program(s) used to refine structure: *SHELXL97* (Sheldrick, 2008 ▶); molecular graphics: *ORTEP-3 for Windows* (Farrugia, 2012 ▶); software used to prepare material for publication: *WinGX* (Farrugia, 2012 ▶) and *PLATON* (Spek, 2009 ▶).

## Supplementary Material

Crystal structure: contains datablock(s) I, global. DOI: 10.1107/S1600536813022629/xu5727sup1.cif

Structure factors: contains datablock(s) I. DOI: 10.1107/S1600536813022629/xu5727Isup2.hkl

Additional supplementary materials:  crystallographic information; 3D view; checkCIF report

## Figures and Tables

**Table 1:** Hydrogen-bond geometry (Å, °)

*D*—H⋯*A*	*D*—H	H⋯*A*	*D*⋯*A*	*D*—H⋯*A*
O2—H2*A*⋯O3^i^	0.82 (3)	2.03 (3)	2.807 (2)	157 (3)
C10—H10*C*⋯O2^ii^	0.98	2.51	3.390 (3)	149

Symmetry codes: (i) 
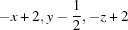
; (ii) 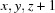.

**Fig. 1.**
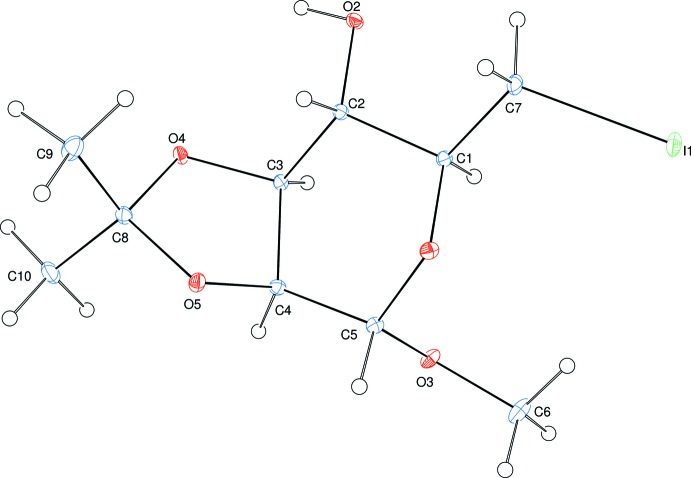
The molecular structure of the title compound, with the atom-numbering scheme. Displacement ellipsoids are drawn at the 50% probability level.

**Fig. 2.**
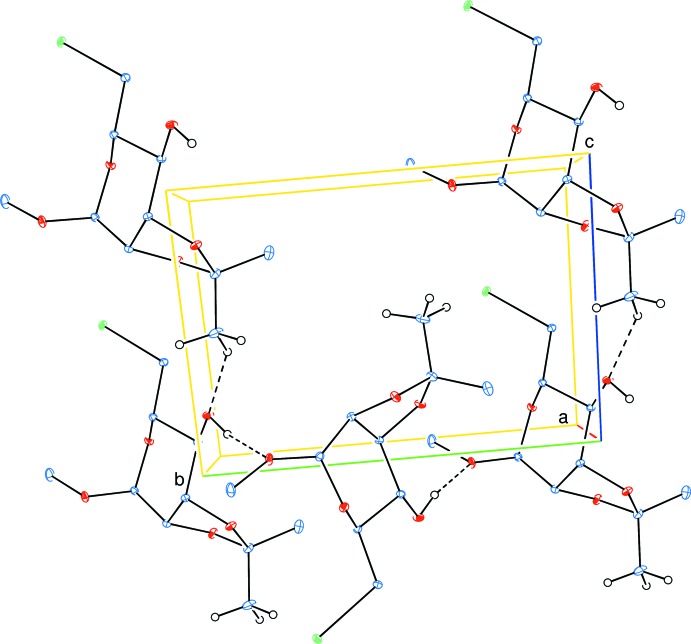
A partial packing diagram. Hydrogen bonds are shown as dashed lines. H atoms not involved in hydrogen bonding have been omitted for clarity.

## supplementary crystallographic information

### 1. Comment

Various carbohydrates have been considerably important for the preparation of unsaturated aldehydes (Kleban *et al.*, 2000; Dransfield *et al.*, 1999; Greul *et al.*, 2001). Conversions of unsaturated aldehydes to oximes (Dransfield *et al.*, 1999), nitrones (Bernet & Vasella, 1979; Greul *et al.*, 2001), nitrile oxides (Gallos *et al.*, 1999; Kleban *et al.*, 2001) and their intramolecular cycloadditions have been reported. These cycloadducts are useful intermediates for the syntheses of polyhydroxylated aminocyclopentane derivatives (Greul *et al.*, 2001; Kleban *et al.*, 2001).

In the title compound (Fig. 1), the ring A (C1–C5/O1) is not planar, but adopts a sofa conformation with puckering parameters (Cremer & Pople, 1975) Q_T_ = 0.551 (2) Å, φ = -113.1 (5)° and θ = 158.5 (2)°. The conformation of ring B (O4/O5/C3/C4/C8) is an envelope, with atom C4 at the flap position, -0.573 (2) Å from the mean plane through the other four atoms. Rings A and B have local pseudo-mirror planes running through C1 and C4 (for ring A), and running through C4 and the midpoint of the O4—C8 bond (for ring B).

In the crystal structure, intermolecular O—H···O and C—H···O hydrogen bonds (Table 1) link the molecules into layers nearly parallel to the *bc* plane (Fig. 2).

### 2. Experimental

The title compound was synthesized in two steps starting from α-D-mannopyranoside by the literature methods (Garegg & Samuelsson, 1980; Bundle *et al.*, 1988; Ichikawa *et al.*, 2004). To a solution of methyl 2,3-*O*-isopropylidene-α-D-mannopyranoside (Evans & Parrish, 1977; Isobe *et al.*, 1981) (2.50 g, 10.66 mmol) in dry toluene (70 ml, dissolved at 353 K) were added PPh_3_ (4.30 g, 15.90 mmol), imidazole (2.17 g, 31.98 mmol) and iodine (3.80 g, 14.90 mmol) sequentially. The reaction mixture was refluxed for 3 h. 20 ml water was added, and then the mixture was extracted with EtOAc (4 × 20 ml). The combined organic phase was washed with brine (300 ml) and then dried over MgSO_4_. The filtrate was concentrated under reduced pressure, and the residue was purified by chromatography (PE:EE 70:30) to afford the iodo compound as a colourless crystalline solid (yield: 90%), m.p. 383–384 K.

### 3. Refinement

Atom H2A (for OH) was located in a difference Fourier map and refined freely. The C-bound H atoms were positioned geometrically, with C—H = 1.00, 0.99 and 0.98 Å for methine, methylene and methyl H atoms, respectively, and constrained to ride on their parent atoms, with *U*_iso_(H) = *kU*_eq_(C), where k = 1.2 for methine and methylene and k = 1.5 for methyl H atoms.

### Crystal data

**Table:**

C_10_H_17_IO_5_	*F*(000) = 340
*M**_r_* = 344.14	*D*_x_ = 1.814 Mg m^−^^3^
Monoclinic, *P*2_1_	Mo *K*α radiation, λ = 0.71073 Å
Hall symbol: P 2yb	Cell parameters from 3798 reflections
*a* = 8.3121 (8) Å	θ = 2.8–30.5°
*b* = 10.3911 (10) Å	µ = 2.55 mm^−^^1^
*c* = 8.3128 (8) Å	*T* = 100 K
β = 118.639 (3)°	Prism, colourless
*V* = 630.15 (11) Å^3^	0.99 × 0.58 × 0.44 mm
*Z* = 2	

### Data collection

**Table:**

Bruker Kappa APEXII DUO diffractometer	3827 independent reflections
Radiation source: fine-focus sealed tube	3803 reflections with *I* > 2σ(*I*)
Triumph monochromator	*R*_int_ = 0.027
φ and ω scans	θ_max_ = 30.5°, θ_min_ = 2.8°
Absorption correction: numerical (Blessing, 1995)	*h* = −11→11
*T*_min_ = 0.187, *T*_max_ = 0.401	*k* = −14→14
13656 measured reflections	*l* = −11→11

### Refinement

**Table:**

Refinement on *F*^2^	Hydrogen site location: inferred from neighbouring sites
Least-squares matrix: full	H atoms treated by a mixture of independent and constrained refinement
*R*[*F*^2^ > 2σ(*F*^2^)] = 0.016	*w* = 1/[σ^2^(*F*_o_^2^) + (0.0159*P*)^2^ + 0.0596*P*] where *P* = (*F*_o_^2^ + 2*F*_c_^2^)/3
*wR*(*F*^2^) = 0.043	(Δ/σ)_max_ = 0.001
*S* = 1.24	Δρ_max_ = 0.86 e Å^−^^3^
3827 reflections	Δρ_min_ = −0.82 e Å^−^^3^
153 parameters	Extinction correction: *SHELXL97* (Sheldrick, 2008), Fc^*^=kFc[1+0.001xFc^2^λ^3^/sin(2θ)]^-1/4^
3 restraints	Extinction coefficient: 0.0949 (17)
Primary atom site location: structure-invariant direct methods	Absolute structure: Flack (1983), 1811 Friedel pairs
Secondary atom site location: difference Fourier map	Absolute structure parameter: 0.003 (12)

### Special details

**Table:**

Geometry. All esds (except the esd in the dihedral angle between two l.s. planes) are estimated using the full covariance matrix. The cell esds are taken into account individually in the estimation of esds in distances, angles and torsion angles; correlations between esds in cell parameters are only used when they are defined by crystal symmetry. An approximate (isotropic) treatment of cell esds is used for estimating esds involving l.s. planes.
Refinement. Refinement of F^2^ against ALL reflections. The weighted R-factor wR and goodness of fit S are based on F^2^, conventional R-factors R are based on F, with F set to zero for negative F^2^. The threshold expression of F^2^ > 2sigma(F^2^) is used only for calculating R-factors(gt) etc. and is not relevant to the choice of reflections for refinement. R-factors based on F^2^ are statistically about twice as large as those based on F, and R-factors based on ALL data will be even larger.

### Fractional atomic coordinates and isotropic or equivalent isotropic displacement parameters (Å^2^)

**Table:**

	*x*	*y*	*z*	*U*_iso_*/*U*_eq_	
I1	0.388710 (12)	0.741920 (17)	0.435425 (11)	0.01675 (5)	
O1	0.59121 (17)	0.64971 (12)	0.87699 (17)	0.0126 (2)	
O2	0.91193 (19)	0.47140 (13)	0.78471 (18)	0.0150 (2)	
H2A	0.998 (4)	0.426 (3)	0.855 (4)	0.045 (10)*	
O3	0.76827 (18)	0.82125 (12)	1.0565 (2)	0.0143 (2)	
O4	1.04540 (17)	0.44287 (13)	1.19016 (17)	0.0130 (2)	
O5	0.80476 (18)	0.50343 (12)	1.23592 (19)	0.0143 (2)	
C1	0.6958 (2)	0.62356 (15)	0.7844 (2)	0.0113 (3)	
H1	0.7689	0.7009	0.7875	0.014*	
C2	0.8224 (2)	0.51098 (16)	0.8849 (2)	0.0111 (3)	
H2	0.7473	0.4379	0.8912	0.013*	
C3	0.9584 (2)	0.55251 (16)	1.0784 (2)	0.0105 (3)	
H3	1.0518	0.6125	1.0768	0.013*	
C4	0.8628 (2)	0.61411 (15)	1.1762 (2)	0.0119 (3)	
H4	0.9530	0.6646	1.2845	0.014*	
C5	0.6980 (3)	0.69768 (17)	1.0559 (2)	0.0118 (3)	
H5	0.6183	0.7038	1.1152	0.014*	
C6	0.6277 (3)	0.91625 (18)	0.9709 (3)	0.0226 (4)	
H6A	0.5518	0.8950	0.8409	0.034*	
H6B	0.6839	1.0010	0.9823	0.034*	
H6C	0.5510	0.9178	1.0309	0.034*	
C7	0.5644 (3)	0.58663 (17)	0.5897 (3)	0.0158 (3)	
H7A	0.4882	0.5136	0.5903	0.019*	
H7B	0.6351	0.5574	0.5287	0.019*	
C8	0.9482 (3)	0.40997 (16)	1.2897 (2)	0.0143 (3)	
C9	0.8614 (3)	0.27863 (19)	1.2334 (3)	0.0220 (4)	
H9A	0.7752	0.2655	1.2809	0.033*	
H9B	0.9570	0.2124	1.2832	0.033*	
H9C	0.7957	0.2727	1.0993	0.033*	
C10	1.0810 (3)	0.4196 (2)	1.4915 (3)	0.0231 (4)	
H10A	1.1313	0.5070	1.5203	0.035*	
H10B	1.1809	0.3579	1.5237	0.035*	
H10C	1.0172	0.4004	1.5618	0.035*	

### Atomic displacement parameters (Å^2^)

**Table:**

	*U*^11^	*U*^22^	*U*^33^	*U*^12^	*U*^13^	*U*^23^
I1	0.01472 (6)	0.01220 (5)	0.01494 (6)	0.00098 (5)	0.00036 (4)	0.00353 (5)
O1	0.0087 (5)	0.0137 (5)	0.0134 (6)	0.0003 (4)	0.0036 (5)	−0.0021 (4)
O2	0.0145 (6)	0.0201 (6)	0.0104 (6)	0.0058 (5)	0.0060 (5)	0.0004 (5)
O3	0.0097 (6)	0.0093 (5)	0.0215 (7)	−0.0009 (4)	0.0055 (5)	−0.0018 (4)
O4	0.0107 (6)	0.0189 (6)	0.0111 (6)	0.0046 (5)	0.0065 (5)	0.0047 (5)
C1	0.0102 (7)	0.0112 (6)	0.0108 (7)	0.0007 (5)	0.0037 (6)	−0.0009 (5)
C2	0.0096 (7)	0.0130 (6)	0.0099 (7)	0.0014 (6)	0.0042 (6)	0.0002 (5)
C3	0.0078 (7)	0.0132 (6)	0.0101 (7)	0.0008 (5)	0.0039 (6)	0.0009 (5)
C4	0.0113 (7)	0.0131 (7)	0.0118 (7)	0.0001 (6)	0.0061 (6)	−0.0006 (6)
C5	0.0102 (7)	0.0116 (6)	0.0125 (8)	−0.0010 (5)	0.0046 (7)	−0.0018 (5)
O5	0.0138 (6)	0.0148 (5)	0.0175 (6)	0.0041 (4)	0.0102 (5)	0.0048 (5)
C6	0.0155 (9)	0.0131 (8)	0.0329 (11)	0.0036 (6)	0.0065 (8)	0.0002 (7)
C7	0.0150 (8)	0.0134 (7)	0.0118 (8)	0.0023 (6)	0.0005 (7)	0.0008 (6)
C8	0.0146 (8)	0.0173 (7)	0.0147 (8)	0.0044 (6)	0.0100 (7)	0.0039 (6)
C9	0.0234 (10)	0.0161 (7)	0.0313 (11)	0.0018 (6)	0.0171 (9)	0.0045 (6)
C10	0.0239 (10)	0.0326 (10)	0.0132 (9)	0.0095 (8)	0.0093 (8)	0.0069 (7)

### Geometric parameters (Å, º)

**Table:**

I1—C7	2.1414 (18)	C4—O5	1.425 (2)
O1—C1	1.4362 (19)	C4—C5	1.522 (3)
O1—C5	1.408 (2)	C4—H4	1.0000
O2—C2	1.4182 (18)	C5—H5	1.0000
O2—H2A	0.823 (16)	C6—H6A	0.9800
O3—C5	1.409 (2)	C6—H6B	0.9800
O3—C6	1.432 (2)	C6—H6C	0.9800
O4—C8	1.4480 (19)	C7—H7A	0.9900
O5—C8	1.433 (2)	C7—H7B	0.9900
C1—C2	1.527 (2)	C8—C9	1.509 (3)
C1—C7	1.505 (2)	C8—C10	1.506 (3)
C1—H1	1.0000	C9—H9A	0.9800
C2—C3	1.519 (2)	C9—H9B	0.9800
C2—H2	1.0000	C9—H9C	0.9800
C3—O4	1.428 (2)	C10—H10A	0.9800
C3—C4	1.524 (2)	C10—H10B	0.9800
C3—H3	1.0000	C10—H10C	0.9800
			
C5—O1—C1	113.31 (13)	O3—C5—H5	108.2
C2—O2—H2A	106 (3)	C4—C5—H5	108.2
C5—O3—C6	112.85 (13)	O3—C6—H6A	109.5
C3—O4—C8	108.25 (12)	O3—C6—H6B	109.5
C4—O5—C8	106.61 (12)	O3—C6—H6C	109.5
O1—C1—C2	106.69 (13)	H6A—C6—H6B	109.5
O1—C1—C7	108.12 (14)	H6A—C6—H6C	109.5
O1—C1—H1	110.5	H6B—C6—H6C	109.5
C2—C1—H1	110.5	I1—C7—H7A	109.0
C7—C1—C2	110.34 (13)	I1—C7—H7B	109.0
C7—C1—H1	110.5	C1—C7—I1	112.76 (11)
O2—C2—C1	108.61 (13)	C1—C7—H7A	109.0
O2—C2—C3	111.71 (13)	C1—C7—H7B	109.0
O2—C2—H2	109.1	H7A—C7—H7B	107.8
C1—C2—H2	109.1	O4—C8—C9	110.52 (14)
C3—C2—C1	109.25 (13)	O4—C8—C10	107.99 (15)
C3—C2—H2	109.1	O5—C8—O4	105.68 (13)
O4—C3—C2	110.49 (14)	O5—C8—C9	108.28 (16)
O4—C3—C4	102.62 (12)	O5—C8—C10	111.02 (14)
O4—C3—H3	110.6	C10—C8—C9	113.10 (16)
C2—C3—C4	111.72 (14)	C8—C9—H9A	109.5
C2—C3—H3	110.6	C8—C9—H9B	109.5
C4—C3—H3	110.6	C8—C9—H9C	109.5
O5—C4—C3	101.37 (12)	H9A—C9—H9B	109.5
O5—C4—C5	109.94 (14)	H9A—C9—H9C	109.5
O5—C4—H4	110.0	H9B—C9—H9C	109.5
C3—C4—H4	110.0	C8—C10—H10A	109.5
C5—C4—C3	115.09 (14)	C8—C10—H10B	109.5
C5—C4—H4	110.0	C8—C10—H10C	109.5
O1—C5—O3	112.13 (14)	H10A—C10—H10B	109.5
O1—C5—C4	113.92 (14)	H10A—C10—H10C	109.5
O1—C5—H5	108.2	H10B—C10—H10C	109.5
O3—C5—C4	106.06 (14)		
			
C5—O1—C1—C2	67.22 (16)	C2—C1—C7—I1	−177.65 (10)
C5—O1—C1—C7	−174.10 (14)	O2—C2—C3—O4	−75.14 (16)
C1—O1—C5—O3	67.12 (17)	O2—C2—C3—C4	171.29 (13)
C1—O1—C5—C4	−53.35 (18)	C1—C2—C3—O4	164.67 (12)
C6—O3—C5—O1	63.69 (19)	C1—C2—C3—C4	51.10 (17)
C6—O3—C5—C4	−171.39 (15)	C2—C3—O4—C8	−95.94 (15)
C3—O4—C8—O5	−0.79 (18)	C4—C3—O4—C8	23.30 (17)
C3—O4—C8—C9	116.14 (17)	O4—C3—C4—O5	−37.10 (16)
C3—O4—C8—C10	−119.67 (15)	O4—C3—C4—C5	−155.67 (14)
C4—O5—C8—O4	−24.18 (18)	C2—C3—C4—O5	81.28 (16)
C4—O5—C8—C9	−142.61 (15)	C2—C3—C4—C5	−37.29 (19)
C4—O5—C8—C10	92.67 (16)	C3—C4—O5—C8	37.73 (17)
O1—C1—C2—O2	172.83 (13)	C5—C4—O5—C8	159.94 (14)
O1—C1—C2—C3	−65.09 (16)	O5—C4—C5—O1	−76.30 (17)
C7—C1—C2—O2	55.61 (18)	O5—C4—C5—O3	159.88 (12)
C7—C1—C2—C3	177.69 (13)	C3—C4—C5—O1	37.36 (19)
O1—C1—C7—I1	66.01 (15)	C3—C4—C5—O3	−86.45 (16)

### Hydrogen-bond geometry (Å, º)

**Table:**

*D*—H···*A*	*D*—H	H···*A*	*D*···*A*	*D*—H···*A*
O2—H2*A*···O3^i^	0.82 (3)	2.03 (3)	2.807 (2)	157 (3)
C10—H10*C*···O2^ii^	0.98	2.51	3.390 (3)	149

Symmetry codes: (i) −*x*+2, *y*−1/2, −*z*+2; (ii) *x*, *y*, *z*+1.

## References

[bb1] Bernet, B. & Vasella, A. (1979). *Helv. Chim. Acta*, **62**, 2401–2410.

[bb2] Blessing, R. H. (1995). *Acta Cryst.* A**51**, 33–38. 10.1107/s0108767394005726 7702794

[bb3] Bruker (2008). *APEX2* and *SAINT* Bruker AXS Inc., Madison, Wisconsin, USA.

[bb4] Bundle, D. R., Gerken, M. & Peters, T. (1988). *Carbohydr. Res.* **174**, 239–251. 10.1016/0008-6215(88)85094-8 2454158

[bb5] Cremer, D. & Pople, J. A. (1975). *J. Am. Chem. Soc.* **97**, 1354–1358.

[bb6] Dransfield, P. J., Moutel, S., Shipman, M. & Sik, V. (1999). *J. Chem. Soc. Perkin Trans. 1*, pp. 3349–3335.

[bb7] Evans, M. E. & Parrish, F. W. (1977). *Carbohydr. Res.* **54**, 105–114.

[bb8] Farrugia, L. J. (2012). *J. Appl. Cryst.* **45**, 849–854.

[bb9] Flack, H. D. (1983). *Acta Cryst.* A**39**, 876–881.

[bb10] Gallos, J. K., Koumbis, A. E., Xiraphaki, V. P., Dellios, C. C. & Coutouli-Argyropoulou, E. (1999). *Tetrahedron*, **55**, 15167–15180.

[bb11] Garegg, P. J. & Samuelsson, B. (1980). *J. Chem. Soc. Perkin Trans. 1*, pp. 2866–2869.

[bb12] Greul, J. N., Kleban, M., Schneider, B., Picasso, S. & Jäger, V. (2001). *ChemBioChem*, pp. 368–370. 10.1002/1439-7633(20010504)2:5<368::AID-CBIC368>3.0.CO;2-A 11828466

[bb13] Ichikawa, Y., Matsukawa, Y., Nishiyama, T. & Isobe, M. (2004). *Eur. J. Org. Chem.* pp. 586–591.

[bb14] Isobe, M., Ichikawa, Y., Kitamura, M. & Goto, T. (1981). *Chem. Lett.* pp. 457–460.

[bb15] Kleban, M., Hilgers, P., Greul, J. N., Kugler, R. D., Li, J., Picasso, S., Vogel, P. & Jäger, V. (2001). *Chembiochem*, pp. 365–368. 10.1002/1439-7633(20010504)2:5<365::AID-CBIC365>3.0.CO;2-M 11828465

[bb16] Kleban, M., Kautz, U., Greul, J. N., Hilgers, P., Kugler, R. D., Dong, H.-Q. & Jäger, V. (2000). *Synthesis*, pp. 1027–1033.

[bb17] Sheldrick, G. M. (2008). *Acta Cryst.* A**64**, 112–122. 10.1107/S0108767307043930 18156677

[bb18] Spek, A. L. (2009). *Acta Cryst.* D**65**, 148–155. 10.1107/S090744490804362X PMC2631630 19171970

